# Formative Evaluation of Safety and Usability of a Mixed-Reality Robot-Assisted Telerehabilitation System for Post-Stroke Upper-Limb Therapy

**DOI:** 10.3390/s26103043

**Published:** 2026-05-12

**Authors:** Md Mahafuzur Rahaman Khan, Kishor Lakshminarayanan, Inga Wang, Jennifer Barber, Erin M. McGonigle Ketchum, Mohammad H. Rahman

**Affiliations:** 1 Department of Mechanical Engineering, University of Wisconsin-Milwaukee, Milwaukee, WI 53211, USA; rahmanmh@uwm.edu; 2 Department of School of Healthcare Science and Engineering, Vellore Institute of Technology, Vellore 632014, India; kishor.ln@vit.ac.in; 3 Department of Rehabilitation Sciences & Technology, University of Wisconsin-Milwaukee, Milwaukee, WI 53211, USA; wang52@uwm.edu; 4 Froedtert Hospital, Milwaukee, WI 53226, USA; jennifer.barber@froedtert.com; 5 Department of Physical Medicine and Rehabilitation, Medical College of Wisconsin, Milwaukee, WI 53226, USA; emcgonigle@mcw.edu

**Keywords:** post-stroke, upper-limb, mixed-reality, telerehabilitation workflow, Internet of Things (IoT), robot-assisted remote therapy, digital twin, HoloLens 2

## Abstract

Robot-assisted telerehabilitation (RAT) combines rehabilitation robotics with digital health workflows to extend access to upper-limb (UL) therapy after stroke. Mixed reality (MR) may support therapist–patient interaction and task visualization; however, early-stage systems require rigorous evaluation of safety and usability before deployment in the home. In a formative, mixed-methods usability study conducted in a controlled setting using a telerehabilitation workflow, six individuals post-stroke (≥3 months) and six occupational therapists (OTs) completed a single supervised session with a desktop-mounted end-effector type therapeutic robot (iTbot) integrated with Microsoft HoloLens 2. Participants performed structured passive and active UL exercises while therapists supervised and interacted with the system via the MR control interfaces. Safety was evaluated by documenting observed adverse events and safety-stop activations. Usability and user experience were assessed using the System Usability Scale (SUS), study-specific satisfaction questionnaires (reported with scale ranges), and semi-structured follow-up interviews analyzed using thematic analysis. All participants completed the session without observed adverse events or safety-stop activations. Overall usability was favorable, with a mean (SD) SUS total score of 78.3 (15.9) out of 100 (stroke: 74.2 [18.1]; occupational therapists: 82.5 [13.5]). Qualitative feedback indicated that MR was perceived as engaging and intuitive by many users, while also identifying implementation needs relevant to real-world telerehabilitation, including clearer onboarding, simplification of certain MR interactions, and improved physical interfaces (e.g., handle options). Therapists highlighted workflow considerations for remote supervision and patient independence. Together, these findings support progression to multi-session, in-home studies to quantify remote assistance needs, technical reliability, adherence, and clinical outcomes.

## 1. Introduction

According to the United Nations, the global aging population will increase from 703 million to 1.5 billion by 2050 [1]. This demographic shift is accompanied by a growing prevalence of age-related disorders such as stroke. Currently, over 50 million people worldwide have experienced a stroke, with more than 13 million new cases occurring annually [2]. Stroke survivors frequently face significant cognitive and physical challenges, including difficulties with concentration, mobility, and the use of their upper limbs (UL) [3].

UL rehabilitation after stroke emphasizes motor relearning through high-repetition, task-specific practice with appropriate progression and feedback.The primary goals of therapists’ customized sessions are to regain proficiency in motor skills and successfully accomplish repetitive activities [4]. In routine care, the dose and intensity of UL training are frequently constrained by therapist availability, insurance coverage, transportation barriers, and the logistical burden of repeated in-person visits [5,6,7,8]. These barriers can contribute to reduced therapy adherence and insufficient practice, particularly for individuals living far from specialized services.

Telerehabilitation has emerged as a strategy to extend rehabilitation services into the home, enabling remote supervision, coaching, and monitoring. Evidence suggests that, for selected post-stroke populations and outcomes, telerehabilitation can achieve clinical results comparable to conventional in-person therapy while improving access and convenience [9,10]. However, many current telerehabilitation approaches remain limited in their ability to deliver the intensity, adaptability, and physical assistance that therapists provide during in-person UL therapy, especially for individuals with moderate-to-severe motor impairment [11]. Robot-assisted therapy (RAT) can address part of this gap by enabling high-repetition practice with programmable assistance or resistance and objective measurement of performance [12]. Despite promising clinical evidence, widespread deployment of UL rehabilitation robots in home-based telerehabilitation remains limited by cost, complexity, workspace requirements, and challenges in delivering intuitive remote therapist interaction and patient feedback [9,13,14]. Consequently, there has been increased emphasis on research into robot-assisted and home-based rehabilitation methods [15,16].

Existing UL telerehabilitation robotic devices, like GERMI [14], MERLIN [9], and Hand Mentor Pro [17], provide focused and limited therapy. Current commercially available therapeutic devices, such as continuous passive motion (CPM) devices, are primarily intended to give passive therapy and do not reproduce the beneficial exercises that therapists do on patients. While numerous commercially available devices, such as ArmeoPower [18], InMotion ARM [19], and ReoGo [20], offer UL rehabilitation, they are primarily intended for clinical usage and do not cover the full spectrum of therapies or mobility required for successful rehabilitation. For example, the InMotion Arm [19] and Rubidium [21] technologies are confined to 2D planar movement, but complete UL rehabilitation needs 3D mobility. These technologies, which are mostly used in clinical settings, are insufficient to cover the full range of UL rehabilitation methods, including joint-based therapy, cartesian position-based therapy, and other types of exercise such as passive, active, and resistive therapy [22,23,24]. As a result, present robotic systems cannot often provide personalized home-based telerehabilitation, which is essential for patients with UL dysfunctions to improve their autonomy and social engagement. Many of these devices are not equipped for telerehabilitation or individualized home-based therapy, which are crucial for expanding access to rehabilitation services. This gap underscores the urgent need for more research into the RAT systems to enhance rehabilitation accessibility, improve patient outcomes, and support the reintegration of stroke survivors into their communities.

Mixed reality (MR) interfaces may improve the usability and acceptability of telerehabilitation robotics by providing intuitive visualization, guided task cues, and interactive feedback. Game-based rehabilitation has also been proposed to support engagement and sustained practice, although claims about motivation and long-term adherence require longitudinal evaluation [25]. In this context, we developed iTbot, a desktop-mounted UL rehabilitation robot integrated with Microsoft HoloLens 2 to deliver guided therapeutic activities and to support therapist interaction through MR-based interfaces. In prior work, the system was characterized from an engineering perspective (e.g., system functionality and performance attributes) [26]. The remaining translational step is to evaluate whether the integrated system can be used safely and effectively by intended end users (stroke survivors and occupational therapists) and whether the workflow is acceptable for future telerehabilitation deployment.

The objective of this study was therefore to conduct a formative, mixed-methods evaluation of the iTbot—HoloLens 2 system with individuals post-stroke and occupational therapists. Participants interacted with an iTbot device to complete a series of therapeutic exercises. This device can provide passive, active, and resistive therapy exercises. Specifically, we assessed (i) observed safety during a supervised session, (ii) usability, and (iii) user experience and workflow acceptability to inform iterative refinement and the design of subsequent multi-session, in-home telerehabilitation trials. These findings provide a foundation for developing experimental setups and formulating study protocols for future clinical trials in home and clinical settings.

## 2. Methodology

### 2.1. Participants Recruitment

Recruitment and ethics: Stroke participants were recruited from the Stroke Survivor Recruitment Database (SSRD) at Froedtert Hospital (Milwaukee, WI, USA). Occupational therapists (OTs) were recruited via institutional and professional networks to provide end-user clinical feedback. The study was approved by the Institutional Review Board (IRB) at the University of Wisconsin-Milwaukee, and all participants provided written informed consent prior to participation.

Study setting and session structure: This study was conducted in a controlled clinical environment using a telerehabilitation workflow (remote monitoring and communication features were available during testing) at BioRobotics Lab. Each participant completed one supervised usability evaluation session that included standardized training and completion of the study tasks with the iTbot—HoloLens 2 system. The session was divided into structured phases, e.g., familiarization and task completion.

Eligibility criteria for stroke participants: Individuals were eligible if they: (1) were ≥18 years old at enrollment; (2) had a radiologically confirmed ischemic or intracerebral hemorrhagic stroke; and (3) were at least 3 months post-stroke at the time of testing (late subacute/chronic phase) to reduce the influence of early spontaneous recovery on performance. Participants were required to have residual UL impairment while retaining sufficient voluntary movement to interact with the system, operationalized as: Fugl–Meyer Assessment–Upper Extremity (FMA-UE) score 10–56 (maximum 66) and Box & Block Test (BBT) performance ≥ 3 blocks/60 s with the affected limb. The FMA-UE and BBT thresholds were selected to (i) avoid floor effects (participants unable to generate any functional movement), (ii) avoid ceiling effects (near-normal UL function), and (iii) ensure participants could safely complete robot-guided and MR-based tasks without excessive assistance.

Exclusion criteria for stroke participants: Participants were excluded if they had conditions that would compromise safe participation or valid assessment, including: severe cognitive impairment limiting informed consent or task comprehension; uncontrolled epilepsy or other conditions with contraindications for head-mounted displays; severe visual impairment not correctable with lenses; severe communication deficits preventing interaction with the study team; or any medical condition of the affected UL that would preclude safe robot-assisted movement.

Eligibility criteria for occupational therapists: OTs were eligible if they had ≥1 year of clinical experience in neurorehabilitation or upper-limb rehabilitation. Therapists were enrolled to evaluate usability, workflow fit, and clinical acceptability of therapist-facing system functions.

### 2.2. Device Description

**Figure 1:**
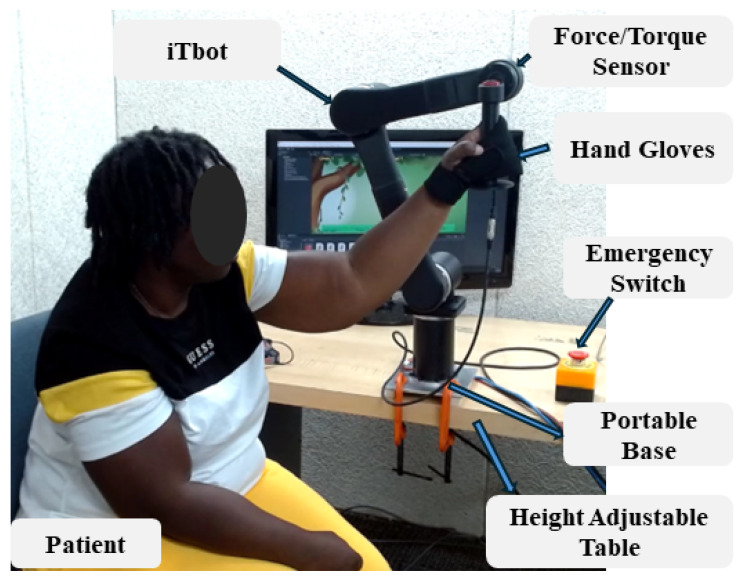
Desktop-mounted intelligent therapeutic robot (iTbot).

An intelligent therapeutic robot (iTbot) is a 3 degrees of freedom (DoF) portable end-effector type serial manipulator, shown in Figure 1, developed as a minimum viable product (MVP) to provide multi-joint UL rehab exercises in 3D space at home. It was fabricated using carbon fiber-reinforced nylon through 3D printing. Each joint of the robot is powered by motors for smooth movement: Joints 1 and 2 use QRob70F [27] motors, while Joint 3 uses a QRob70H [28] motor. A 6 axis force/torque sensor is instrumented underneath the robot’s wrist handle to detect human–robot interactive force and maneuver it accordingly based on the rehab exercise. Additionally, the rehab robot requires a 48 V power supply and EtherCAT communication cables compatible with the TwinCAT3 [29] system. It can provide passive, active, and resistive therapy.

Safety Features of iTbot: iTbot was designed with comprehensive safety features to protect both patients and therapists. Hardware safety features include adjustable mechanical stoppers for personalized joint limits; software safety features are added in the controller that include limiting the joints’ ranges of movements depending on the participant’s requirements and limiting the joints’ speed, torques, and voltage values, which are the final output of the controller and the command values to the motor. Three emergency switches (for the patient, therapist, and researcher) are installed to cut off the power if necessary. The system also stops at the point of inadvertent contact with the subject and therapist.

### 2.3. iTbot Telerehabilitation System

**Figure 2:**
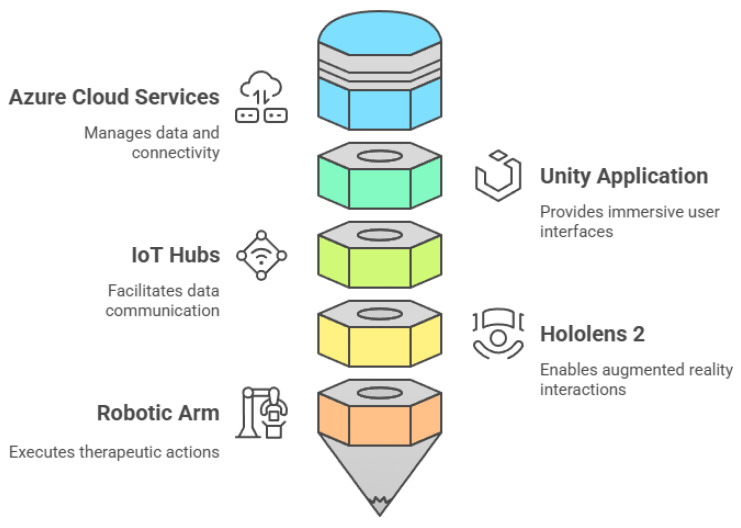
Data flow diagram illustrating the integration of a robotic rehabilitation system with a telerehabilitation platform.

The iTbot telerehabilitation system is an advanced therapeutic platform designed for remote rehabilitation, leveraging cutting-edge technologies for precision and real-time control [26]. The system’s architecture is built on the Microsoft Azure platform [30] for robust cloud computing, Unity for immersive user interfaces, and the TwinCAT3 [29] system for seamless software integration, as shown in Figure 2.

A digital twin mimics the physical robot in real-time, giving therapists precise and dynamic feedback by allowing them to monitor real-time data, including movement metrics and robot-generated parameters, through a secure, connected system powered by Azure IoT. The Azure IoT platform facilitates continuous data exchange, enabling responsive interactions between system components via the Internet. Human–machine interaction is achieved through an MR-based controller integrated within a Unity application, accessible via Microsoft HoloLens 2 [31]. This interface allows operators to manipulate the robot’s state, with state changes transmitted through Azure IoT Hub to a PC that commands the iTbot. The system architecture includes Azure storage for data logging and multiple IoT hubs for precise command execution and operational data relay. This ensures real-time monitoring and control of the robotic system for a highly engaging therapeutic experience.

### 2.4. Experimental Setup

**Figure 3:**
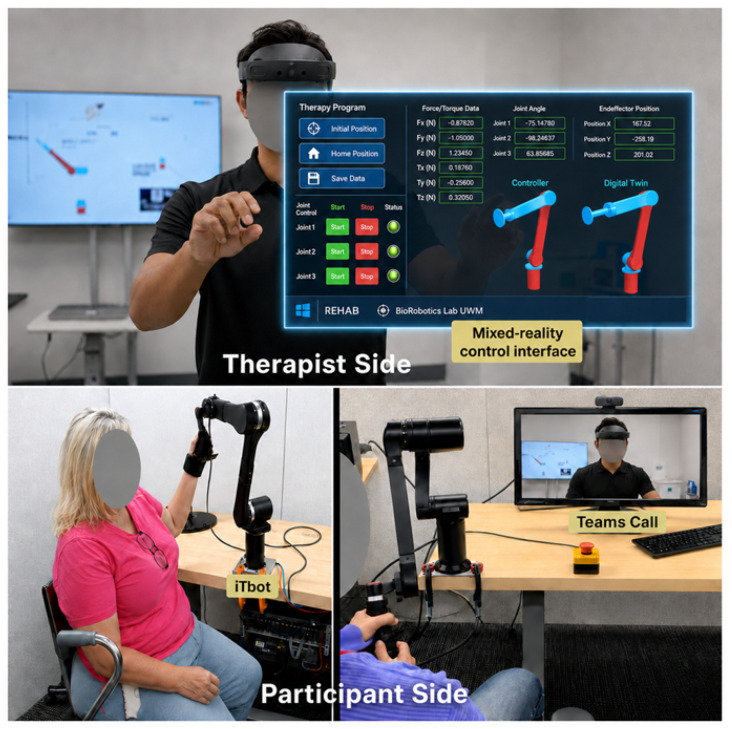
Two-station experimental setup simulating a telerehabilitation workflow. The upper panel shows the therapist side, where the occupational therapist uses Microsoft HoloLens 2 to interact with the MR-based control interface while monitoring the participant via a live Microsoft Teams video feed. The lower panel shows the participant side, where the stroke participant is seated at the desktop-mounted iTbot robot, grasps the wrist handle, and has access to the emergency stop switch.

The evaluation used a two-station configuration consisting of a therapist/operator station and a participant station, as shown in Figure 3. The study was conducted in a controlled environment while using a telerehabilitation-style workflow (video communication and cloud-based monitoring/control). The therapist and participant were located in the BioRobotic Lab, UWM, in separate rooms, with supervision provided via Microsoft Teams.

The therapist interacted with the system using a Microsoft HoloLens 2 mixed-reality (MR) interface that displayed robot status and therapist-facing controls for configuring passive therapy parameters within predefined safety limits (Figure 3, “Therapist Side”). The therapist maintained continuous visual and verbal contact with the participant using a live Teams video feed displayed on a standard monitor at the therapist’s station.

Participants were seated at the iTbot with standardized posture and alignment and grasped the handle(s) during robot-mediated tasks (Figure 3, “Participant Side”). A camera and audio setup enabled continuous two-way communication with the therapist via Teams. Throughout the session, the iTbot recorded kinematic and interaction data (e.g., end-effector position/velocity and interaction forces/torques). These data were streamed in real time through the Azure cloud to the therapist-facing interface to support monitoring and parameter adjustment during the supervised session.

**Figure 4:**
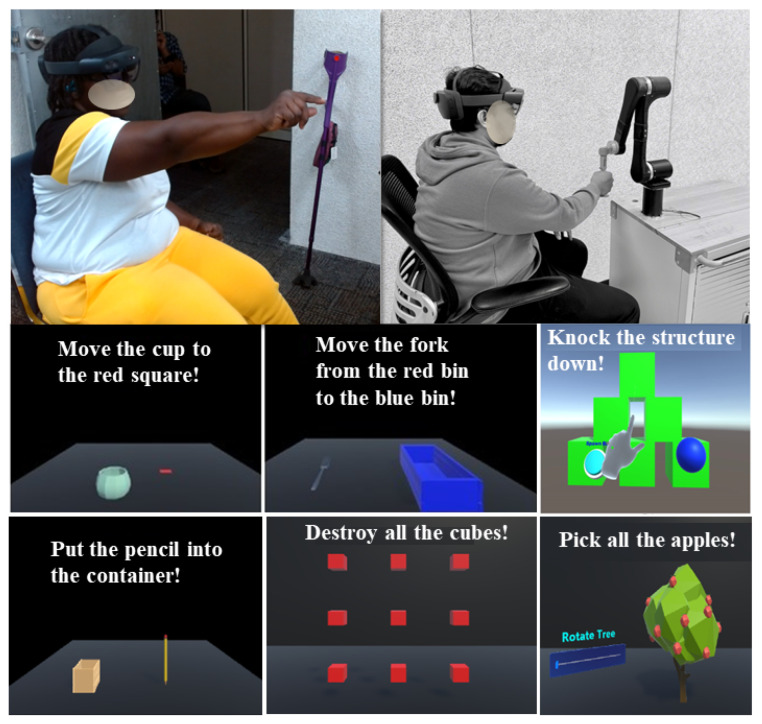
Participants engaging in active therapy through gamified levels using the HoloLens 2.

Two therapy modes were evaluated:

- Passive therapy (robot-driven movement). The therapist used the MR interface to guide the iTbot through slow, controlled trajectories tailored to each participant’s tolerated range of motion. The interface enabled real-time adjustment of movement parameters, including speed, direction, and trajectory selection, within predefined safety constraints. Participants completed three therapist-guided robot trajectories: shoulder flexion/extension, horizontal abduction/adduction, and a combined reaching pattern. Each trajectory was performed for at least 10 movement cycles (repetitions) at a standardized slow speed of approximately 10°/s, with therapist adjustment permitted within predefined safety limits. This resulted in approximately 25 min of passive therapy time-on-task.
- Active therapy (participant-driven tasks with feedback). Participants performed goal-directed UL tasks designed around motor-learning principles [32]. MR content provided task cues and performance feedback through gamified levels (Figure 4). Depending on the task, active therapy was delivered either with the iTbot, involving active/resistive robot interaction with therapist-adjusted resistance based on participant performance and tolerance, or without the iTbot, involving MR-only tasks. Participants completed six gamified MR task levels involving goal-directed object manipulation tasks that increased in complexity. These tasks were time-based rather than repetition-based; each level lasted approximately 3–5 min, resulting in approximately 30 min of active therapy time-on-task. Rest breaks were permitted as needed to minimize fatigue. The total session duration, including onboarding, task completion, questionnaires, and follow-up interview, was approximately 90–120 min per participant. The present study focused on usability, user experience, and workflow feasibility rather than long-term adherence or clinical efficacy.

Overall, the system combined therapist supervision via video communication, therapist configuration via MR, and real-time monitoring of robot-interaction signals to support a supervised telerehabilitation-style workflow during the usability evaluation.

### 2.5. Assessments and Procedure

**Figure 5:**
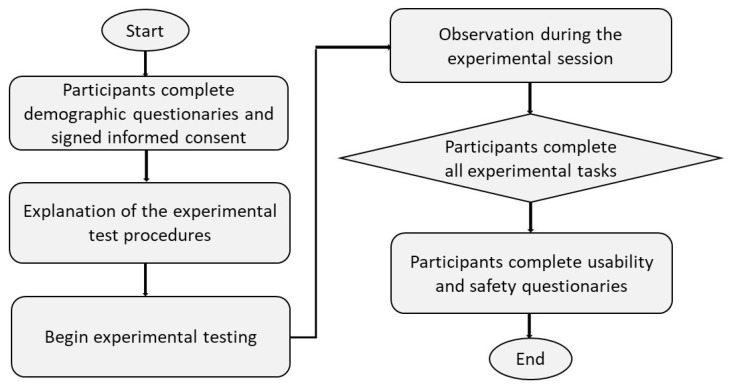
Flowchart depicting the sequence of a usability test process.

The evaluation was designed as a formative, mixed-methods usability assessment to characterize observed safety, usability, and user experience of the iTbot–HoloLens 2 system and to identify implementation issues relevant to future multi-session, in-home studies. Figure 5 summarizes the study workflow.

Session procedure: Each participant completed one supervised session consisting of (1) onboarding and familiarization with the iTbot and the HoloLens 2 interface, (2) completion of standardized passive and active tasks (robot-guided and MR-supported activities as described in Section 2.4), and (3) completion of questionnaires followed by a semi-structured interview. Research staff observed all tasks and documented usability issues using a standardized observation form. Audio/video recordings were collected to support qualitative analysis of user feedback and to verify observational notes.

Safety assessment: Safety was assessed by documenting observed adverse events (e.g., pain, discomfort, excessive fatigue, dizziness, skin irritation), task discontinuations, and any safety-stop or emergency-stop events. Robot telemetry (e.g., end-effector position, velocity, and interaction forces and torques) was continuously monitored during testing to support safe task execution and was logged for post hoc review.

Usability and user experience measures: After completing the session tasks, participants completed: (1) the System Usability Scale [33] (SUS; 10 items, 5-point Likert; scored 0–100, higher scores indicate better usability); (2) study-specific questionnaires assessing perceived engagement, gamification and satisfaction with key system elements (e.g., clarity of tasks, perceived feedback, comfort, and interface usability), with response options and score ranges reported in the Supplement; and (3) an experience evaluation survey assessing perceived usefulness and workflow fit. Occupational therapists completed therapist-facing versions of the questionnaires focusing on clinical workflow, safety, and feasibility of remote supervision, whereas stroke participants completed participant-facing versions emphasizing comfort, clarity, and perceived usability.

Quantitative analysis: Questionnaire outcomes were summarized using descriptive statistics (mean ± SD or median [IQR], as appropriate) and visualized with individual data points due to small sample size. Between-group comparisons (stroke vs. OT) were treated as exploratory and performed using independent-samples *t*-tests. Statistical significance was interpreted cautiously given the pilot nature of the study and limited power.

Sample size rationale: This study was designed as a formative usability evaluation to identify usability problems, implementation barriers, and design priorities for iterative refinement, rather than to estimate clinical effect sizes or test treatment efficacy. Therefore, the study did not require the sample size expected for summative validation or clinical trials. This approach is consistent with FDA human factors guidance [34], IEC 62366-1:2015 [35], and established usability problem-discovery models [36,37]. Using the Nielsen–Landauer model, $P\left(n\right)=1-{\left(1-p\right)}^{n}$, with an individual problem-detection probability of p=0.31, a sample of n=6 identifies approximately 88% of discoverable usability problems [36]. Thus, the present sample (n=6 stroke participants and n=6 occupational therapists) was selected to capture patient and clinician perspectives as distinct stakeholder groups. This sample size is also consistent with published formative usability evaluations of rehabilitation technologies [26,38]. Between-group comparisons were treated as exploratory and interpreted cautiously. The findings are intended to guide system refinement and inform the design of future multi-session, in-home studies with larger and more diverse cohorts.

Qualitative analysis: Semi-structured interview data and observation notes were analyzed using thematic analysis. Two researchers independently coded notes, reconciled discrepancies by consensus, and generated themes. Themes were reported separately for stroke participants and OTs to distinguish patient experience from clinical workflow considerations.

## 3. Results

### 3.1. Participant Characteristics

Twelve participants completed the evaluation: six individuals post-stroke (SP1–SP6) and six occupational therapists (OT1–OT6). The system was assessed using questionnaires encompassing multiple dimensions, including usability, gamification, satisfaction, and overall experience with the rehabilitation device. Demographic characteristics are summarized in Table 1.

### 3.2. System Usability Score (SUS)

Usability was assessed using the System Usability Scale [33,39] (SUS; total score range 0–100), summarized in Table 2. It demonstrates that both stroke participants (SP1–SP6) and therapist participants (TP1–TP6) generally rated the system positively across key usability dimensions. Stroke participants experienced slightly more challenges in adapting to the system’s interface compared to OTs, who consistently found the system intuitive and user-friendly. The mean (SD) SUS total score was 74.17 (18.14) for stroke participants and 82.50 (13.51) for OTs (overall: 78.33 (15.86)). Item-level SUS responses are provided in Table 2 for transparency. Stroke participants reported higher ratings on items reflecting support needs (e.g., technical assistance), whereas OTs reported uniformly high ratings for ease of use and learnability (Table 2).

### 3.3. Gamification Questionnaire Engagement and Motivation Period

**Figure 6:**
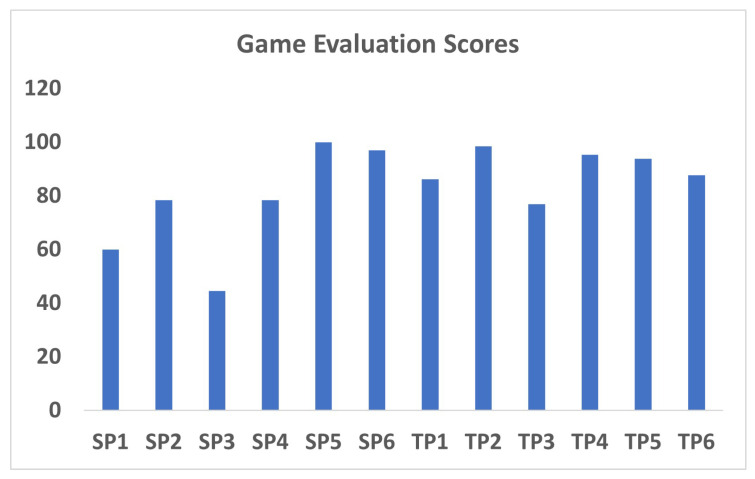
Game evaluation individual scores.

The gamification elements [40] of the system (Table 3) were well rated by both groups, specifically the clear objectives, an engaging environment, and intuitive feedback mechanisms (Figure 6). The clarity of goals and tasks was strongly rated, as participants consistently felt that they understood what was expected of them during interactions with the system. All OTs rated the interface as more user-friendly than stroke participants, suggesting that minor adjustments could improve accessibility for individuals with motor or cognitive impairments. However, stroke participants reported slightly lower scores for the variety of exercises and their adaptability, indicating a need for greater personalization of tasks to align with individual rehabilitation goals.

### 3.4. Weighted System Evaluation (Functionality, Reliability, Usability, Adoption)

A weighted evaluation matrix [41] was used to summarize perceived system performance across four domains: Functionality (max 350), Reliability (max 200), Usability (max 100), and Adoption (max 200), computed by multiplying each item response (Likert 1–5) by its assigned weight and summing within each domain.

The domain scores represent participants’ perceived evaluation of the system during the supervised usability session and should not be interpreted as objective measures of clinical effectiveness or long-term system performance. Participants did not directly select categorical labels such as “Significantly Functional,” “Highly Functional,” “Significantly Reliable,” or “Highly Reliable.” Instead, participants rated individual items within each domain on a 1–5 Likert scale, and the corresponding categorical level was assigned by the researchers based on predefined score ranges from the weighted evaluation rubric.

The Functionality Score (FS) was derived from items assessing perceived suitability, flexibility, usefulness, accuracy, interoperability, transparency, real-world fit, and perceived efficacy. The Functionality Level (FL) was then assigned based on the computed FS. Similarly, the Reliability Level (RL), Usability Level (UL), and Adoption Level (AL) were derived from their respective weighted domain scores. Reliability in this evaluation refers specifically to perceived system reliability during the supervised usability session. The Reliability Score (RS) was calculated from items assessing perceived system maturity, operational stability, security, and safety. Each item was multiplied by its assigned weight and summed to generate the RS, with a maximum possible score of 200. Objective long-term reliability metrics, such as connectivity interruptions, hardware/software faults, and sensor dropouts, were not primary outcomes in this formative study and will be evaluated in future multi-session in-home trials.

Mean (SD) domain scores were as follows: Functionality: stroke 316.67 (35.73), OT 319.17 (29.57); Reliability: stroke 171.67 (22.29), OT 190.00 (6.32); Usability: stroke 81.67 (16.33), OT 88.33 (14.38); Adoption: stroke 163.33 (14.38), OT 172.50 (14.40). Based on the rubric thresholds, most participants were categorized as highly functional (10/12), highly reliable (11/12), highly usable or significantly usable (11/12), and highly adoptable or significantly adoptable (12/12).

### 3.5. Safety and Satisfaction Questionnaire [41] Outcomes

**Figure 7:**
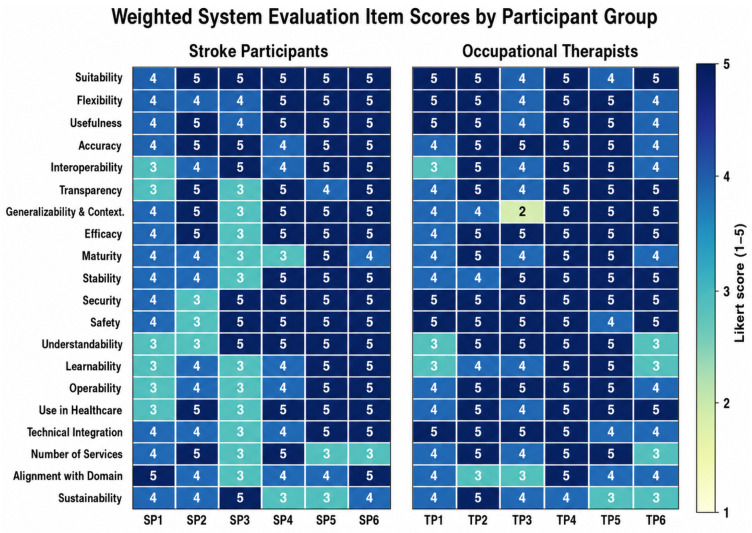
Individual satisfaction item scores. Each cell shows the raw 1–5 Likert score for a given evaluation item, with darker shading indicating higher ratings.

The satisfaction scores reflected the level of confidence in the system’s ability to support rehabilitation. Both groups rated the system’s suitability, flexibility, and safety highly, with OTs generally rating these aspects higher than stroke participants (Figure 7). The system’s ability to produce reliable and accurate results was rated well, although stroke participants indicated that there was room for improvement in transparency and technical integration. Despite these challenges, both groups found the system emotionally engaging and easy to use once they had adapted to its controls (Table 4).

Safety and satisfaction items were rated on a 1–5 Likert scale (higher scores indicate more favorable ratings). Both groups rated contact safety and assembly safety highly (Table 5). Stroke participants reported lower ratings for control-related items (e.g., operational intuitiveness and adjustability) compared with OTs (Table 5). Composite means (SD) by domain were: Safety: stroke 4.39 (0.90), OT 4.67 (0.51); Emotional satisfaction: stroke 4.53 (0.48), OT 4.69 (0.45); Efficiency satisfaction: stroke 4.25 (0.76), OT 4.42 (0.58); Control satisfaction: stroke 4.00 (0.89), OT 4.75 (0.61).

### 3.6. Observed Safety Outcomes

The safety outcomes (Table 5) demonstrated that participants felt secure using the device, with high ratings for contact safety, assembly safety, and operational safety. Both groups expressed satisfaction with the safety features of the system, including the presence of emergency protocols. All participants completed the protocol. No adverse events were observed during testing (e.g., no pain requiring discontinuation, no skin injury, and no headset intolerance leading to termination). No emergency-stop activations occurred. Post-session safety-related ratings are summarized in Table 5.

### 3.7. Participants Feedback

Qualitative feedback was collected from semi-structured interviews, observational notes, and session recordings to capture user experience and to identify design priorities for future iterations. To improve clarity, feedback is summarized by theme and separated for stroke participants and occupational therapists (OTs).

Stroke participants (SP1–SP6): Theme 1—Perceived benefit of robot-guided passive stretching: Several participants highlighted passive stretching as a valued component of the session (e.g., SP1: “The stretching exercises are especially helpful for my arm.”).

Theme 2—Convenience and interest in future home use: Some participants expressed that a remotely supported model could reduce the burden of traveling for therapy (e.g., SP2: “Since I prefer staying indoors, it’s great that I don’t have to go out to get therapy.”). Because this study was conducted in a controlled setting, these comments are interpreted as preferences for future home deployment rather than evidence of at-home feasibility.

Theme 3—Ergonomics and accessibility needs: Participants frequently noted the need for improved physical interfaces, particularly handle designs suitable for spasticity or limited grasp (e.g., SP2: “The vertical handle is difficult to hold…A horizontal handle would make it easier for me to grip.”). Portability and setup ease were also raised (e.g., SP4: “A table with wheels would make it easier to move the device around.”).

Theme 4—Display preferences for active tasks: Preferences varied regarding the head-mounted display. Some participants requested an alternative 2D display option due to visual needs or comfort concerns (e.g., SP2: “A larger 2D screen would help me see the game content more clearly,” and SP3: “I don’t want to wear the head-mounted display.”). These comments suggest that offering both HoloLens-based and 2D display modes may improve accessibility.

Theme 5—Engagement with goal-directed tasks: Participants reported that task-based interactions could encourage movement during the session (e.g., SP5/SP6: “It really motivates me to move, especially when I can place something in a basket.”). These statements reflect perceived engagement during a single evaluation session and do not imply sustained adherence or clinical efficacy.

Occupational therapists (OT1–OT6): Most of the OTs emphasized practical needs for future in-home implementation, including how setup would be managed for individuals with limited arm movement (e.g., donning gloves and attaching the end-effector), the need for multiple handle/forearm-support options matched to impairment level, and the importance of detecting intention-to-move and resistance direction to individualize resistance. OTs also recommended simplifying and staging MR task design (e.g., starting with achievable 1D tasks and progressing to 2D tasks with graded difficulty) to reduce frustration and support completion.

## 4. Discussion

This formative, mixed-methods study evaluated observed safety, usability, and user experience of the iTbot–HoloLens 2 telerehabilitation workflow in a controlled setting. Three main findings emerged. First, no adverse events were observed during supervised use and participants rated safety-related aspects highly, supporting the adequacy of the current safety design for controlled evaluation. Second, usability ratings were favorable overall, with System Usability Scale (SUS) total scores averaging 74.2/100 for stroke participants and 82.5/100 for occupational therapists (OTs), indicating good perceived usability with variability across individuals. Third, qualitative feedback identified implementation priorities for future in-home deployment, particularly related to physical interfaces (handles/attachments), display accessibility (HoloLens vs. 2D screen options), and support needs.

Telerehabilitation has been proposed to improve access [43] to post-stroke rehabilitation by reducing travel burden and extending therapist reach, while rehabilitation robotics can increase practice dose [44] and provide programmable assistance and resistance with objective measurement. However, real-world adoption of home-based robotic telerehabilitation remains limited by setup burden, usability constraints, and the need for reliable remote supervision and technical support [45]. In this context, the present study provides early evidence that an integrated robot–MR system can be operated in a supervised telerehabilitation-style workflow with favorable usability and safety perceptions, while also revealing practical barriers that must be resolved before broader home deployment. A key translational insight is the distinction between workflow feasibility and home independence. Stroke participants reported higher agreement on SUS items reflecting support needs (e.g., requiring technical assistance and needing more background information). These responses suggest that, in its current form, the system may not yet be appropriate for fully independent use by all stroke survivors without additional support. This finding motivates targeted refinements, including simplified user flows, structured onboarding with competency checks, caregiver-assisted setup options for users with limited UL movement, and remote technical support pathways.

The iTbot and HoloLens 2 serve complementary roles. The iTbot provides the physical human–robot interaction required for passive and active upper-limb training, enabling programmable motion guidance and measurement of interaction signals. The HoloLens 2 provides a mixed-reality (MR) interface layer used to support therapist-facing configuration and monitoring and to present task cues and feedback for MR-supported activities. In the present study, therapist involvement remained central for safety monitoring, parameter adjustment, and coaching via video communication. Therefore, the findings should not be interpreted as demonstrating autonomous home training. Rather, they support the feasibility of a supervised workflow and motivate future work to determine which components can be safely transitioned toward greater patient independence (e.g., preconfigured exercise plans, automated safety checks, and simplified MR interactions) without increasing risk.

User interface (UI) design and accessibility emerged as important determinants of user experience. While many participants reported that MR content was engaging, others preferred a larger 2D display or did not wish to wear a head-mounted display for extended use. These findings support offering alternative display modes (HoloLens 2 and 2D screen), adjustable visual parameters (e.g., font size and target salience), and task designs that minimize headset burden. In parallel, multiple stroke participants and OTs emphasized the need for modular handle and attachment options, including forearm-support designs, to accommodate spasticity, limited grasp, and varying impairment levels. These considerations are particularly relevant for telerehabilitation, where challenging setup or poor fit can reduce adherence and may increase safety risk.

This study has several strengths, including the involvement of both intended end-user groups (stroke survivors and OTs), the use of mixed methods to capture quantitative usability and safety ratings alongside qualitative workflow insights, and improved visualization of the therapist-facing MR interface to enhance interpretability. Nevertheless, the findings should be interpreted in light of several limitations. First, the sample size was small (n = 6 per group) and not representative, as all stroke participants were female, which limits generalizability. Second, the evaluation was conducted in a controlled setting rather than in participants’ homes; therefore, common telerehabilitation challenges, including connectivity issues, remote troubleshooting, caregiver availability, device storage constraints, and longitudinal adherence, were not captured. Third, the study assessed perceived usability, acceptability, and observed safety during supervised sessions but did not evaluate clinical efficacy, dose effects, or sustained motivation, which require multi-session longitudinal trials with functional outcome measures. Fourth, several study-specific questionnaires were used; while appropriate for formative evaluation, future work should prioritize validated implementation and user-experience instruments where available and report reliability metrics for any custom tools. Finally, spasticity was not formally quantified using a validated scale such as the Modified Ashworth Scale; therefore, the influence of spasticity severity on usability, handle tolerance, and robot interaction could not be systematically evaluated. Although several participants reported or demonstrated grip-related difficulty that informed the qualitative design recommendations, future multi-session studies should include standardized neurological characterization, including spasticity grading, FMA-UE, BBT, and detailed impairment profiles.

The results motivate a staged evaluation pathway. Next steps include multi-session studies in the home environment to quantify: (i) frequency and reasons for remote assistance (technical issues, setup, symptom management), (ii) system reliability metrics (connectivity, sensor faults, task interruptions), (iii) adherence and dose delivered, (iv) safety events and near-misses using predefined adverse event criteria, and (v) preliminary clinical outcomes (e.g., FMA-UE and functional task measures) over a clinically meaningful period. In parallel, hardware and UI refinements should target attachment/handle modularity, reduced setup burden, and dual-mode display support (MR and 2D screen) to maximize accessibility across visual and comfort needs. As telerehabilitation continues to evolve, the iTbot may contribute to future telerehabilitation workflows if confirmed in multi-session in-home studies.

## 5. Conclusions

This formative, mixed-methods evaluation indicates that the iTbot–HoloLens 2 system can be implemented within a supervised, controlled telerehabilitation-style workflow without observed adverse events and with favorable usability and acceptability ratings from both stroke participants and occupational therapists. These findings support further development and staged evaluation of the platform for upper-limb telerehabilitation, while underscoring that additional refinement is needed before independent home use is appropriate for all users. Priorities include streamlined onboarding and remote support, impairment-tailored attachment/handle options (e.g., forearm support for limited grasp or spasticity), and accessible display alternatives (HoloLens 2 and 2D screen modes). Future studies should include larger and more diverse cohorts and extend to multi-session, in-home deployments to quantify remote assistance needs, technical reliability, adherence, safety events, and preliminary clinical outcomes over clinically meaningful durations.

## Figures and Tables

**Table 1:** Demographic data of the participants.

Participant ID	Employment Status	Age	Gender	Affected Limb	Stroke Onset (mm/yyyy)
SP1	E	57	F	L	07/2024
SP2	R	76	F	R	03/2016
SP3	R	70	F	L	04/2021
SP4	U	43	F	L	10/2007
SP5	R	56	F	R	07/2003
SP6	R	63	F	R	12/2005
TP1	OT	29	F	–	–
TP2	OT	20	F	–	–
TP3	OT	23	M	–	–
TP4	OT	20	F	–	–
TP5	OT	42	F	–	–
TP6	OT	54	F	–	–

Abbreviations: Participant ID: SP = Stroke Participant, TP = Therapist Participant; Employment Status: E = Employed, U = Unemployed, R = Retired, OT = Occupational Therapist; Gender: M = Male, F = Female; Affected Limb: R = Right, L = Left.

**Table 2:** SUS scale questions include the mean (M), standard deviation (SD), t-statistic, and *p*-value for each item, comparing responses from all stroke participants and OT individuals.

Item	Question	Stroke M ± SD	OT M ± SD	t-Statistic	*p*-Value
Q1	I would like to use this robot/system often	4.33 ± 0.82	4.83 ± 0.41	−1.34	0.20
Q2	I think this robot/system is complex to use	2.17 ± 1.33	2.00 ± 1.55	0.20	0.84
Q3	I think this robot/system is easy to use	4.17 ± 0.98	5.00 ± 0.00	−2.07	0.06
Q4	I required technical assistance to use this robot/system	3.50 ± 1.38	2.33 ± 1.63	1.33	0.21
Q5	I think the functionalities of this robot/system are well integrated	4.50 ± 0.55	4.83 ± 0.41	−1.19	0.26
Q6	I think the functionalities of this robot/system are not consistent	1.83 ± 1.33	1.67 ± 1.51	0.19	0.84
Q7	I think most users can quickly learn to use this robot/system	4.50 ± 0.84	4.83 ± 0.41	−0.87	0.40
Q8	I think most users have difficulties learning to use this robot/system	1.83 ± 1.33	2.00 ± 1.41	−0.21	0.83
Q9	I am confident when using this robot/system	4.17 ± 0.98	4.83 ± 0.41	−1.53	0.15
Q10	I need to learn more background information about this robot/system before use	2.67 ± 1.63	2.67 ± 2.25	0.00	0.99

**Table 3:** Gamification Questionnaire with mean ± standard deviation scores of all the participants.

Criteria	Questionaries	Rank
Game	1. Objective/goal <Unclear (1) → Clear (5)>	4.16 ± 0.98
	2. Level of difficulty <Low (1) → High (5)>	3.00 ± 1.26
	3. Ignorance of achievement <Unawareness (1) → Awareness (5)>	3.83 ± 1.60
	4. Environment <Unattractive (1) → Attractive (5)>	4.33 ± 0.81
	5. User Interface <Not user-friendly (1) → User-friendly (5)>	4.00 ± 1.54
	6. Beginning and end <Unclear (1) → Clear (5)>	4.00 ± 1.67
Exercises	7. Instructions <Unclear (1) → Clear (5)>	3.66 ± 1.63
	8. Variation <Low (1) → High (5)>	3.50 ± 1.51
	9. Suitable for game goal <Low (1) → High (5)>	3.33 ± 1.86
	10. Feedback <Unclear (1) → Clear (5)>	4.16 ± 0.98
User	11. Motivating challenge <Low (1) → High (5)>	4.33 ± 1.21
	12. Mistake permission <Impossible (1) → Possible (5)>	3.50 ± 1.64
	13. Security feeling <Uncomfortable (1) → Comfortable (5)>	4.00 ± 1.54

**Table 4:** Satisfaction Questionnaire Scores, including stroke participants and OTs [42].

ID	FS	FL	RS	RL	US	UL	AS	AL
SP1	260	SF	160	HR	60	U	155	SA
SP2	335	HF	140	SR	75	SU	175	HA
SP3	285	HF	160	HR	70	SU	140	SA
SP4	330	HF	180	HR	85	HU	165	HA
SP5	340	HF	200	HR	100	HU	165	HA
SP6	350	HF	190	HR	100	HU	180	HA
TP1	295	HF	180	HR	70	SU	170	HA
TP2	340	HF	190	HR	95	HU	190	HA
TP3	275	SF	190	HR	95	HU	165	HA
TP4	350	HF	200	HR	100	HU	190	HA
TP5	340	HF	190	HR	100	HU	165	HA
TP6	315	HF	190	HR	70	HU	155	SA

Abbreviations: FS = Functionality Score; FL = Functionality Level; RS = Reliability Score; RL = Reliability Level; US = Usability Score; UL = Usability Level; AS = Adoption Score; AL = Adoption Level. Domain levels were derived from weighted score ranges in the evaluation rubric; participants rated individual items on a 5-point Likert scale and did not directly select the categorical levels.

**Table 5:** Classification and Evaluation Scores (M ± SD) of iTbot.

Classification	Definition	Scores
Contact Safety	When using the device, the part that comes in contact with the user’s body does not injure the skin (pain, redness, rash, swelling, or bleeding where the skin is rubbed off).	4.50 ± 0.83
Assembly Safety	The assembly is solid and sturdy.	4.50 ± 0.83
	The subject’s body can be easily fixed to the device.	4.16 ± 0.98
Operational Safety (Electric)	It is easy to check the operation status, such as power on.	4.33 ± 1.03
Satisfaction Safety	I am satisfied with the system’s safety features.	4.33 ± 1.03
	There is an emergency button that is used in an emergency.	4.50 ± 0.83
Emotional Satisfaction	The size is appropriate.	4.50 ± 0.54
	The texture is good.	4.50 ± 0.54
	The color is good.	4.50 ± 0.54
	The design is good.	4.33 ± 0.51
	The device is stable.	4.66 ± 0.51
	The noise is not loud (during operation).	4.66 ± 0.51
Efficiency Satisfaction	The device is easy to use.	4.50 ± 0.83
	It is easy to set up the device for exercise.	4.00 ± 0.89
Control Satisfaction	Operation is intuitive.	4.00 ± 0.89
	It is easy to adjust the height or the length according to the subject’s body shape.	4.00 ± 0.89

## Data Availability

The datasets used and analyzed in this study are available upon reasonable request from Md Mahafuzur Rahaman Khan (khan45@uwm.edu).
